# The role of proteomics and radioproteomics in biomarkers of liver HCC for diagnosis, therapy and enhance interventional radiological treatments

**DOI:** 10.15761/crr.1000244

**Published:** 2025-06-05

**Authors:** Margaret Simonian

**Affiliations:** David Geffen School of Medicine, Department of Radiological Sciences, University of California Los Angeles, California, United States

**Keywords:** liver cancer, proteomics, biomarkers, hepatocellular carcinoma

## Abstract

Hepatocellular carcinoma (HCC) is the most common liver cancer and is highly malignant. Current diagnostic tests are blood, imaging and biopsy, with no useful predictive biomarker. Additionally, targeted therapies are unavailable, which hinders the potential for personalized therapy in HCC patients. Here, we discuss the limited studies conducted thus far to identify molecular targets for early detection and treatment of HCC, their limitations and future directions in this field. However, the integration of multi-omics analyses, such as genomics, proteomics, and phosphoproteomics, has provided valuable insights into the mechanisms and pathways underlying the disease.

## Introduction

### Liver Hepatocellular Carcinoma (HCC):

Liver HCC is the most common type of primary liver cancer, originating in the hepatocytes, the liver’s main functional cells. It is a major global health concern, with incidence rates rising due to factors like chronic liver diseases, including hepatitis B and C infections, liver cirrhosis, and non-alcoholic fatty liver disease (NAFLD). As the liver plays a crucial role in detoxification, metabolism, and protein synthesis, the onset of HCC can severely impair these functions, leading to significant morbidity and mortality [1,2].

The pathogenesis of HCC is multifactorial, often beginning with chronic liver injury. Chronic viral infections, particularly hepatitis B and C, are the leading causes of HCC worldwide. Hepatitis C virus (HCV) and hepatitis B virus (HBV) infections cause long-term liver inflammation, leading to fibrosis and cirrhosis, which are major risk factors for the development of HCC. Other major contributors include alcohol abuse, which leads to alcoholic liver disease and cirrhosis, as well as NAFLD, which is becoming increasingly common due to rising obesity rates [3]. Genetic mutations, such as those in the tumor suppressor gene p53, and altered signalling pathways, like the Wnt/β-catenin pathway, have been identified in the progression of HCC [4].

Early-stage HCC often presents without noticeable symptoms, which makes it difficult to detect until it has advanced. When symptoms do appear, they may include weight loss, jaundice (yellowing of the skin and eyes), abdominal pain, a swollen abdomen, and fatigue. These symptoms often overlap with those of other liver diseases, making diagnosis challenging without appropriate testing.

To diagnose HCC, imaging techniques such as ultrasound, computed tomography (CT), and magnetic resonance imaging (MRI) are commonly used to visualize liver lesions. A biopsy, can help confirm the diagnosis, and blood serum markers, including alpha-fetoprotein (AFP), are often elevated in HCC. While AFP levels can be indicative of liver cancer, they are not entirely reliable for early detection or for monitoring disease recurrence.

## Treatment options

Treatment for HCC depends on several factors, including the stage of the cancer, the underlying liver function, and whether the patient has cirrhosis. Early-stage HCC may be treated with surgical options, such as liver resection or liver transplantation. Liver transplantation is particularly effective for patients with cirrhosis, as it removes both the tumor and the underlying diseased liver.

For patients who are not candidates for surgery, locoregional treatments may be employed. These include techniques like radiofrequency ablation (RFA), transarterial chemoembolization (TACE), and percutaneous ethanol injection. These treatments aim to destroy the tumor cells or block their blood supply without removing the liver. In more advanced cases of HCC, systemic therapies like targeted therapies and immunotherapy are becoming increasingly important. Sorafenib, a tyrosine kinase inhibitor, is one of the most commonly used targeted therapies. Immunotherapy agents, such as immune checkpoint inhibitors (e.g., nivolumab and pembrolizumab), have shown promise in the treatment of advanced HCC by boosting the body’s immune system to fight cancer cells [5–8].

The prognosis for HCC varies widely depending on the stage at diagnosis and the treatment provided. Early detection and intervention is the key to improving survival rates. However, due to the aggressive nature of the disease and its tendency to be diagnosed at advanced stages, the overall prognosis remains poor, particularly in patients with underlying liver cirrhosis [9,10].

## Proteomics in HCC biomarker studies

Proteomics, the large-scale study of proteins, plays a critical role in understanding the molecular mechanisms underlying diseases like HCC. Proteins are directly responsible for carrying out the biological functions encoded by the genome, and they often undergo post-translational modifications (PTMs) that are crucial for their function and regulation [11,12]. The dynamic nature of protein expression and modification in HCC makes proteomics an essential tool for identifying biomarkers that can serve as diagnostic or prognostic indicators.

Very few proteomics have been attempted, and very few proteomics biomarkers have been studied for liver HCC, and their correlation to clinical behaviour and response to therapy is limited. The lack of well-defined targetable drugs also limits the potential of personalized treatment for HCC patients [2].

Liver HCC is very heterogeneous, and currently there are no targeted therapy used to treat patients based on their genetic mutations [5]. Therefore, protein profiling may provide potential targets when combined with patient’s imaging data (radioproteomics) and genomics data (proteogenomics). Furthermore, mass spectrometry-based proteomics may reveal additional biological insights that cannot be achieved by imaging or genomics data alone, because the changes at genomic and transcriptomic level not always are translated into proteins, and post-translational modifications such as protein phosphorylation which is necessary in regulating protein activity are mostly missed and could not be represented by genomic profiling alone [13].

Additionally, proteomics may help in understanding how HCC develops at the molecular level, particularly the pathways involved in hepatocyte transformation, such as the Wnt/β-catenin pathway, epithelial-to-mesenchymal transition (EMT), and inflammatory signalling. By identifying proteins involved in these pathways, proteomics could not only aid in early diagnosis but also provide potential targets for therapy, contributing to the development of more personalized treatment strategies, particularly when combined with radiological imaging data (Radioproteomics), a rapidly emerging field.

**Figure 1.**
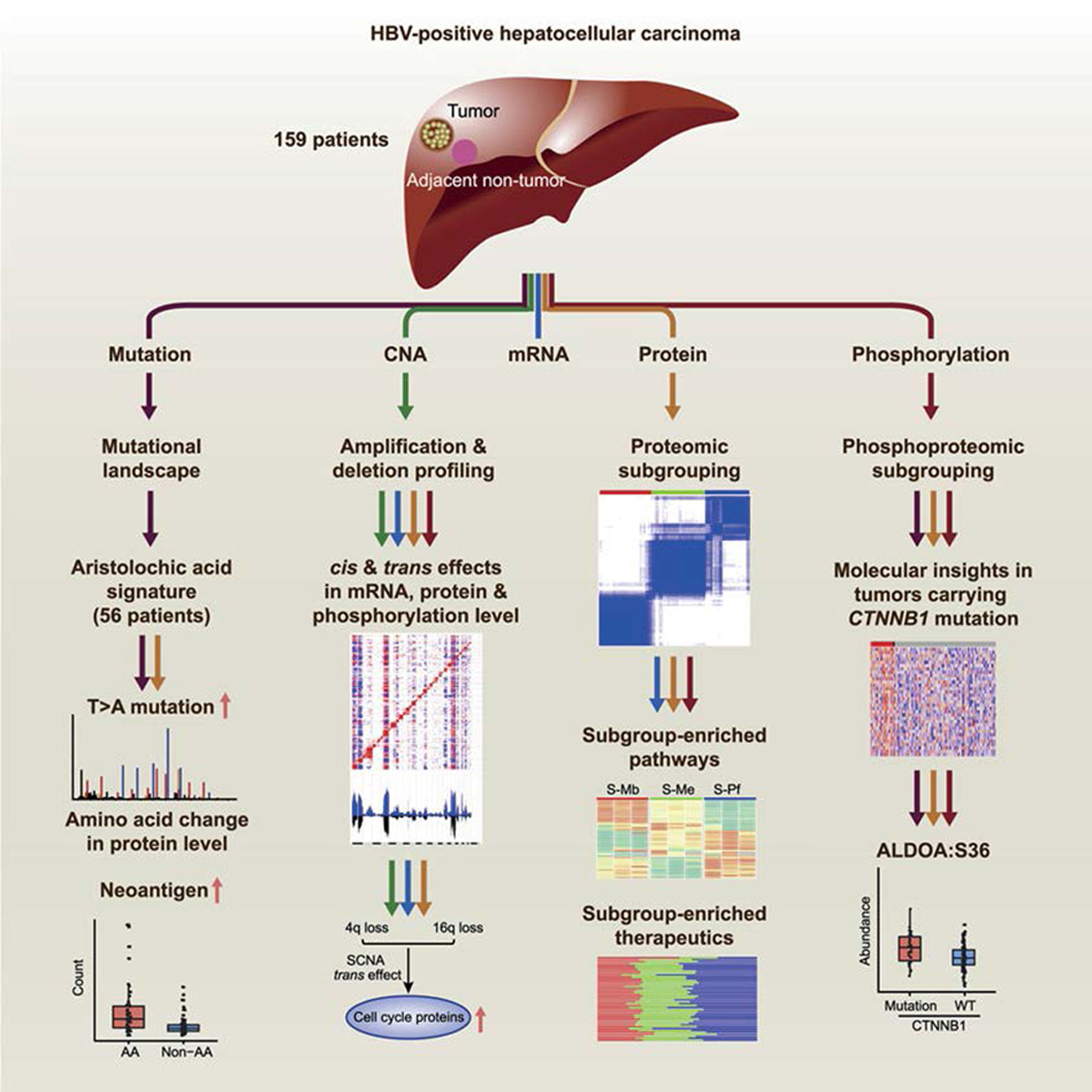
Proteogenomic workflow of the liver HBV related HCC on 159 patients [14]

Gao, et al. [14], performed proteogenomic study of hepatitis B virus related liver HCC, on 159 patients using paired tumor and adjacent liver tissues (Figure 1). The whole exome sequencing analysis identified 10,235 mutated genes, including 20,369 non silent point mutations and 1,363 small insertions deletions. And the phosphoproteomics identified 59,746 phosphosites from 9,224 phosphoproteins with average 28,401 phosphosites per sample. Two metabolic enzymes, PYCR2 and ADH1A, were also identified as potential prognostic biomarkers and the subsequent omics analyses revealed five mutated genes TP53 (58%), CTNNB1 (19%), AXIN1 (18%), KEAP1 (7%), and RB1 (6%).

Although this study suggested that metabolic alterations are perhaps the most important factor associated with advanced disease stage, and that targeting cancer metabolism may potentially be used as a treatment for HCC [14], the study only focused on HBV related HCC, excluding other causes such as chronic hep C, cirrhosis and heavy alcohol use.

**Figure 2.**
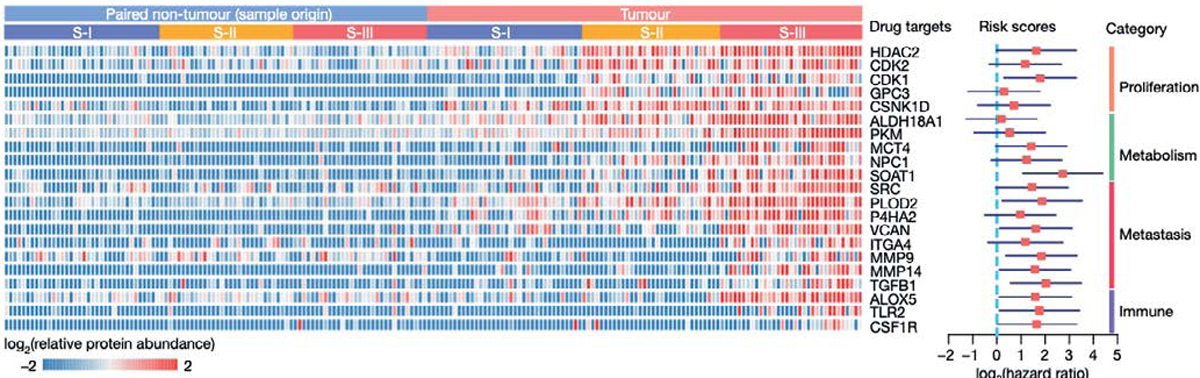
Potential drug targets for HBV-related early-stage HCC [15]

Another phosphoproteomics study by Jiang, et al. [15], conducted on early-stage HBV related HCC of 110 patients who had no chemotherapy or radiotherapy, classified three subtypes each with a different clinical outcome (S-I, S-II and S-III). Patients with S-III subtype was associated with the lowest survival rate and the greatest risk of a poor prognosis after surgery, expressed high levels of SOAT1 protein in the tumour tissues compare to adjacent non-tumour liver tissues. The tissue microarray (TMA) assay also showed upregulation of SOAT1 (which catalysis the formation of fatty acid-cholesterol esters); therefore, they suggested that targeting this protein may prove beneficial in future clinical therapy (Figure 2). Previous studies have demonstrated that elevated SOAT1 expression is linked to poor prognosis in various cancers, including prostate and pancreatic cancer [16,17]. This suggests that high SOAT1 expression could be a common characteristic across multiple types of cancer and may serve as a potential prognostic biomarker and therapeutic target for several tumours, including HCC.

**Figure 3.**
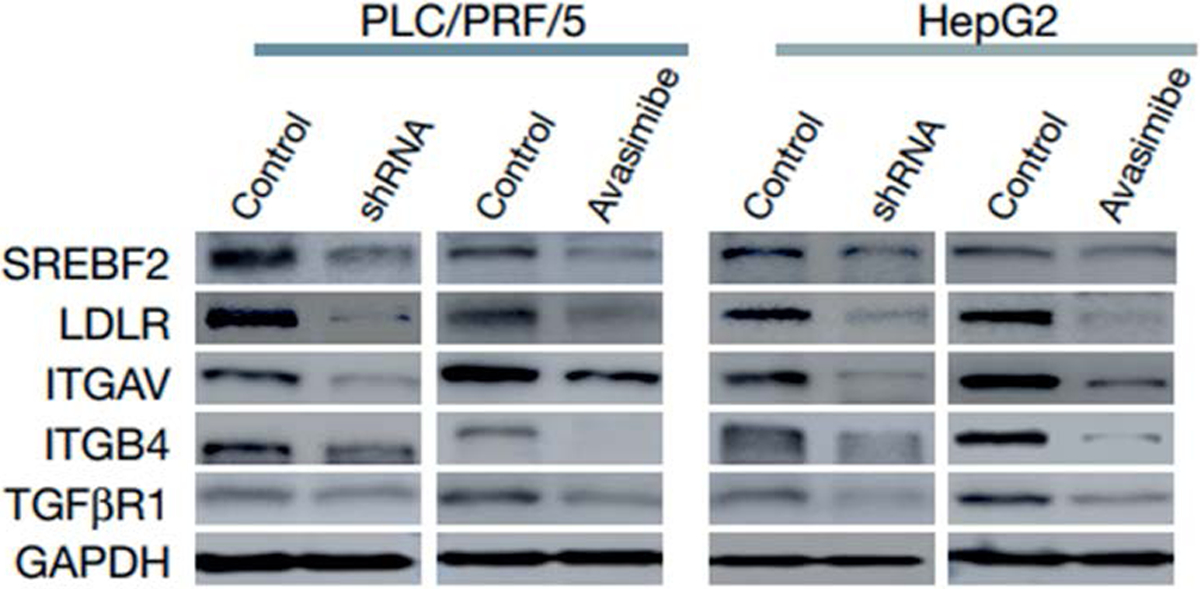
Immunoblot validation of Integrins and TGFβR1 by SOAT1 knockdown and avasimibe treatment [15]

To confirm the role of SAOT1, they knocked down SOAT1 in two HCC cell lines and treated them with avasimibe which is SOAT1 inhibitor. The proteomics analysis of these cell lines, post knockdown and treatment, showed that multiple receptors (intergrins and TGFβ) located on the plasma membrane which are crucial for tumour growth and metastasis, were significantly decreased (P < 0.05, at least 1.5-fold). Integrins were one of those receptors. Since they are regulated functionally by the cholesterol level of the plasma membrane, the SOAT1 knockdown and treatment with avasimibe reduced the cholesterol levels of the plasma membrane of the tumour cells, hence they concluded that downregulation of SOAT1 suppresses the proliferation and migration of HCC cells by reducing the cholesterol content of the plasma membrane, and then inhibiting the integrin and TGFβ signalling pathways (Figure 3).

In 2018, a quantitative proteomics pilot study on HCC tissue biopsies from patients who underwent liver transplant, identified differentially expressed proteins in poor-differentiated, moderate-differentiated, well-differentiated tissues. Over 4000 proteins were identified, with 35 proteins up-regulated (> 3-fold) in advanced tumors compare to controls (Table 1) [18]. More recently, in 2024, a study led by Simonian, et al. [19], on paired FFPE and frozen tissue (FF) percutaneous core biopsies of liver (LIRADS 5) of varying histological grades, identified differentially expressed proteins and genes, in poor, moderate and well-differentiated HCC tissues.

**Figure 4.**
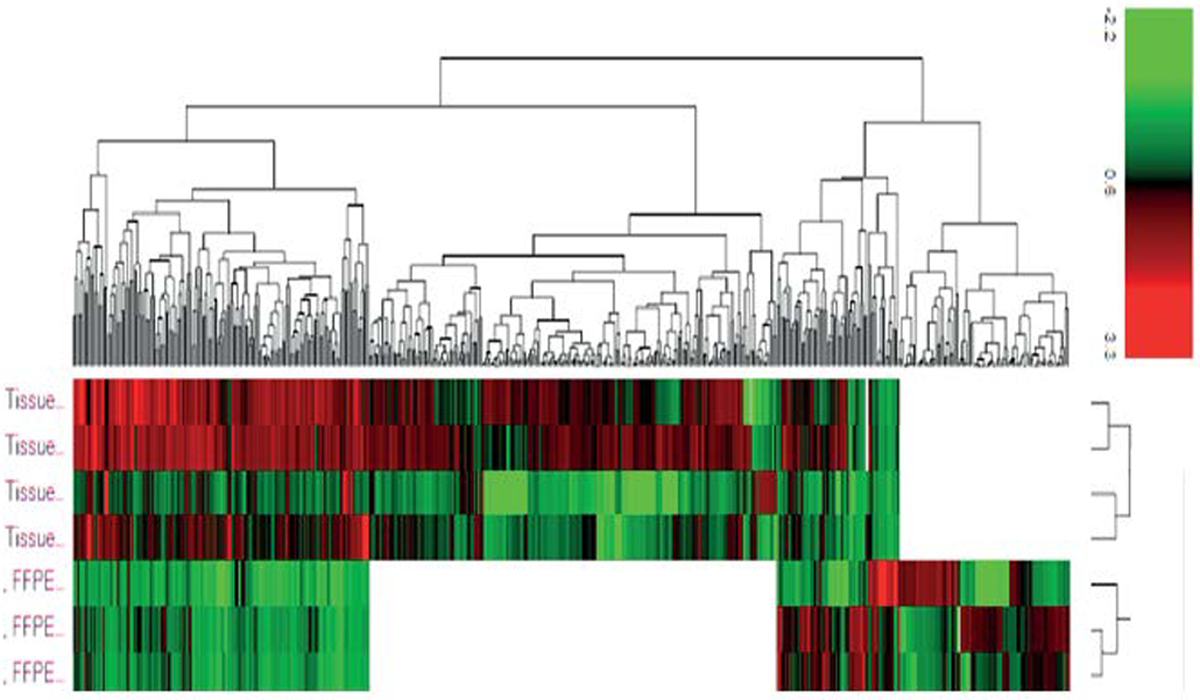
Heatmap of all protein identified in FF and FFPE of liver HCC from proteomics analysis [19]

The proteomics data identified 222 overlapping differentially expressed proteins between FFPE and FF. More upregulated proteins were identified in FF compared to FFPE is all phenotypes, with greater fold-change in FF, due to higher concentration of proteins in frozen tissues (Figure 4). Within overlapping proteins comparison analysis, many proteins were upregulated in moderate compared to well-differentiated tissue cores, in both FF and FFPE, with a greater fold change in FF, such as CES-1, Profilin-1, PDI, Vimentin and more [19]. Additionally, 195 proteins were upregulated in poor *vs.* moderate-differentiated tissues in FF, and 214 proteins were upregulated in poor *vs.* well-differentiated in FF (Table 2).

While the RNA-Seq data identified 594 overlapping genes, 5 of which were significantly upregulated (fold-change >2) in moderate *vs.* well differentiated tissue in both FF and FFPE, with greater fold change in FF samples e.g., MBL2 expression in (moderate FF) *vs.* (well FF) = 25-fold, while in (moderate FFPE *vs.* (well FFPE) = 3-fold; GLUL expression in (moderate FF) *vs.* (well FF) = 27-fold, while in (moderate FFPE *vs.* (well FFPE) = 5-fold (Table 3).

Many of the genes and proteins identified in this study are involved in cancer progression, cell proliferation, and immune response. By integrating radiomics, radiogenomics, and radioproteomics, they were able to provide valuable insights into the characteristics and potential suitability of HCC core tissues for future research. This approach enhances the understanding of the molecular foundations of HCC across various radiological classifications, which may ultimately improve clinical decision-making and patient outcomes [20–22].

## Conclusion

While the proteomic studies mentioned above have demonstrated the feasibility and importance of using proteomics for liver HCC, more extensive quantitative global proteomics research is needed with larger patient cohorts. This should include tissue and serum samples from HCC patients with diverse etiological backgrounds, not limited to HBV, to gain a deeper understanding of HCC tumors for early detection and the development of personalized medicine. Furthermore, the protein candidates identified in some of these studies have not been validated in independent patient cohorts and lacked thorough follow-up, which significantly limits our understanding of their clinical significance and translational potential. While treatments for HCC are advancing, early detection remains crucial to improving survival rates. As research continues to explore new therapies and preventive measures, hope remains for better outcomes for patients suffering from this aggressive form of cancer.

## Figures and Tables

**Table 1.** Some of the differentially expressed proteins in poor, moderate and well differentiated HCC and control tissues

Proteins	Poor *vs.* C	Well *vs.* C	Poor *vs.* Well	Proteins	Poor *vs.* C	Well *vs.* C	Poor *vs.* Well
VIME	3.1	2.3	1.4	MUC18	3.1	2.3	1.3
ANXA2	3.6	2.5	1.4	IBP1	3.1	2	1.6
COEA1	2.7	2.2	1.2	MMAB	2.2	1.7	1.3
IGKC	3.2	2.5	1.3	S10AA	3	2.3	1.3
CSPG2	3.1	2.1	1.4	ANGL6	2.4	1.9	1.2
PALLD	2.8	1.9	1.4	LAMB1	3.1	2.4	1.3
MVP	2.8	2.2	1.2	NIPS2	2.5	1.9	1.3

**Table 2.** Some of the upregulated proteins >1 fold, in (poor *vs.* moderate) and (poor *vs.* well) differentiated in frozen tissues FF

Protein name	Fold-change	Fold-change
	poor *vs.* moderate	poor *vs.* well
Aldehyde dehydrogenase	1.34	1.53
Profilin-1	1.01	1.23
Actin-related protein	1.15	1.11
Isoform of P0DMV9, Heat shock 70	1.12	1.12
Alpha-actinin-4	1.32	1.35
Isoform of P32754	1.1	1.72
Catalase	1	1.98
Adenosyl homocysteinase	1.07	1.04
Serine hydroxymethyl transferase	1.49	1.74
Apolipoprotein A-I	1.44	1.17
Glycine amidino transferase	1.43	2.9
Myosin-9	1.02	1.06
Protein disulfide-isomerase A6	1.07	1.13
Sulfotransferase 1A1	1.22	2.27
Endoplasmin	1.18	1.03
Protein disulfide-isomerase	1.01	0.93
Isoform of P06737, Alpha-1,4	1.13	1.41

**Table 3.** The significantly upregulated genes >2 fold, in moderate *vs.* well differentiated tissue cores in both FF and FFPE, with greater fold change in FF, due to higher gene counts

Gene name	Normalized gene count moderate	Normalized gene count well	Fold-change (moderate *vs.* well)	Normalized gene count moderate	Normalized gene count well	Fold-change (moderate *vs.* well)
SCD	3280.54	101.16	32.41	1503.52	535.03	2.81
ACSL4	929.61	7.07	131.55	181.51	32.86	5.52
MBL2	392.09	15.94	24.58	194.96	65.80	2.96
RELN	596.45	24.03	24.81	293.06	81.29	3.61
GLUL	8238.2	300.55	27.41	4000.74	840.19	4.76
